# Effect of hit rate and cognitive style on Bayesian reasoning: evidence from eye movements

**DOI:** 10.3389/fpsyg.2025.1485283

**Published:** 2025-03-17

**Authors:** Lin Yin, Zifu Shi, Mei Liu, Huohong Chen

**Affiliations:** Cognition and Human Behavior Key Laboratory of Hunan Province, School of Educational Science, Hunan Normal University, Changsha, China

**Keywords:** Bayesian reasoning, breast cancer problem, eye movement, cognitive style, hit rate

## Abstract

While psychological research has established both probability information and cognitive style as key factors in Bayesian reasoning, their interactive effects remain underexplored. We conducted an eye-tracking experiment with 52 undergraduates using EyeLink II to examine how hit rate variations and field dependence/independence influence reasoning patterns during classic Bayesian tasks. Results revealed significant hit rate × cognitive style interactions across multiple eye-tracking measures (total/average fixation durations, area-specific dwell time, fixation proportion). The attention prioritization followed the order: hit rate > false alarm rate > base rate, though base rate information retained measurable influence. High hit rates amplified field-dependent participants’ base rate neglect, while field-independent individuals maintained stable attention allocation across conditions. Field-independent reasoners demonstrated superior concentration and more efficient cognitive resource allocation, employing systematic information-processing strategies. These findings clarify the cognitive hierarchy of probability weighting in Bayesian reasoning while validating the critical moderating role of individual differences in information processing styles.

## Introduction

People frequently rely on their reasoning and decision-making abilities, and the outcomes of these processes significantly impact decision-makers. Prior to making decisions, individuals gather relevant information and engage in reasoning based on the acquired information and their existing knowledge.

Bayesian reasoning involves the adjustment and integration of existing evidence and prior knowledge to estimate the subjective probability of inverse conditional events (Shi et al., 2019). Since Edwards initiated the study of Bayesian reasoning in 1968 (Edwards, 1968), numerous scholars have examined this topic, exploring information representation, mosaic sets, and individual factors, among other aspects, resulting in a wealth of research findings (Gigerenzer and Hoffrage, 1995; Cohen and Staub, 2015). These investigations align with dual-process theories that distinguish intuitive heuristics (System 1) from analytical processing (System 2), particularly in how individuals weigh base rates versus vivid likelihoods. More recently, researchers have begun to delve into the reasoning process itself (Johnson and Elisabet, 2015; Kolossa et al., 2015; Kopp et al., 2016; Reani et al., 2017; Shi et al., 2019).

In a study conducted by Zhang et al. (2003), they manipulated the level of base rates and their position in statements, further analyzing the phenomenon of base rate neglect based on Kahneman’s work. The results showed that variations in base rate significantly influenced participants’ posterior probability estimates. The study investigated the relative importance of different probabilities and found that the order of importance was as follows: hit rate, base rate, and false alarm rate. In terms of inference judgments, participants did not confuse the hit rate with posterior probability, highlighting the hit rate as the primary factor in their decision-making. Adjustments to the hit rate were made using it as the reference point, while also considering the base rate and false alarm rate.

This tension between statistical norms and cognitive strategies underscores the need to examine stable individual differences. Cognitive style, as an essential personality trait, plays a crucial role in influencing reasoning abilities (Tang and Shi, 2011). They used disease-related problems as reasoning materials to investigate the impact of cognitive style on Bayesian reasoning performance when the nested set relationships in the reasoning tasks were clearly defined, found that field-independent participants and field-dependent participants demonstrate contrasting information search strategies. During the process of solving Bayesian reasoning problems, field-independent individuals’ tendency to disembed from context may facilitate System 2-type analytical processing of base rates, while field-dependent individuals tend to rely on the given problem situation and are restricted to the provided information (Shi et al., 2010).

Wang and Ouyang (2004) used the embedded figures test to classify university students into field-independent and field-dependent types. Under a segmented time-limited condition, participants solved the B, C, D, and E group problems from the Raven’s Progressive Matrices. The results revealed that cognitive style is a significant personality variable that impacts the reasoning proficiency of college students. They found that field-independent individuals outperformed their field-dependent counterparts in terms of reasoning performance. Additionally, they discovered differences in the cognitive processing styles and the difficulty levels of the problems. As the complexity of reasoning problems increases, the advantages of field-independence become more pronounced, suggesting field-independent individuals may better engage analytical systems when managing complex probabilistic relationships.

Previous research on the cognitive mechanisms of Bayesian reasoning has mostly employed text-based paradigms, using posterior probability estimates, response times, and other indirect indicators to infer the information gathering and integration processes within Bayesian reasoning. In recent years, eye tracking technology has been widely used to study visually related higher cognitive activities, such as reasoning, and has yielded a series of valuable findings. For example, Bai et al. (2008) analyzed total fixation time and gaze revisit counts, finding that the process of linear syllogistic reasoning involved both linguistic processing and representational processing. Shimojima and Katagiri (2013) used eye tracking to provide real-time and detailed data metrics for diagrammatic reasoning within a limited space. Building on these advances, the current study integrates dual-process theory with cognitive style assessment to employ eye tracking technology to quantify individual reasoning processes in real-time data, shedding light on the role of base rate information in Bayesian reasoning.

## Methods

### Participants

This experiment received approval from the Ethics Committee of Hunan Normal University in China, and all participants provided written informed consent before taking part. The sample consisted of 52 college students in Changsha (Reani et al., 2017), including 20 males and 32 females, with average ages of 25 and 20 years, respectively. All participants were second-year students with no background in medicine and no prior knowledge of Bayes’ theorem, Chinese native speakers and normal vision or corrected to normal vision.

### Experimental design

This experiment was a 3 (hit rate: high: 80% / middle: 50% / low: 10%) × 2 (cognitive style: field-dependent / field-independent) between-subjects design.

The dependent variables were a series of eye movement indexes, including total fixation time, number of fixations, average fixation time, pupil diameter changes and various eye movement indexes in the interest area. Among these variables, total fixation time refers to the sum of all the fixation times of a reader in a certain area, the number of fixation times refers to the number of fixations falling in a certain area, and the average fixation time refers to the average time spent by a reader each time he or she makes a fixation. In this study, the change in pupil diameter is the operational definition, and it is also the most commonly used index in the study of eye movement. Pupil diameter distinguishes the psychological load of the subjects, but the pupil diameter of an individual varies greatly. Only investigating the difference in pupil diameter may have an impact on the analysis due to noise related to individual differences. Moreover, this study aims to explore the psychological load change in the whole reasoning process; thus, the whole reasoning process is divided into 20 equal parts, on average, and the average pupil diameter in the first part is used as the baseline. The variation in pupil diameter in this study refers to the difference between the average pupil diameter and the baseline.

## Materials and instruments

### Embedded figures test (EFT)

The EFT was administered to classify participants as field-dependent or field-independent. The test required participants to identify simple geometric shapes (e.g., triangles, rectangles) embedded within complex visual patterns within a 10-min time limit. Each correct identification earned 1 point, with a maximum score of 20. Participants were classified as field-independent if their scores fell above the sample mean (mean score = 12.3, SD = 3.1) and field-dependent if below. This approach aligns with established protocols (Salmani Nodoushan, 2007; Zhang, 2004), where higher scores indicate stronger ability to disembed details from contextual distractions.

### Bayesian reasoning task

The task utilized a modified breast cancer screening problem [adapted from Tang and Shi (2011)].

The context paradigm problem was employed to conduct Bayesian inference, with a base rate of 1% and a false alarm rate of 9.5%. Consistent with previous studies (Tang and Shi, 2011), the high hit rate was set at 80% while the low hit rate was set at 10%. The problem materials utilized identical wording and sentence structure.

### Area of interest

**Figure 1:**
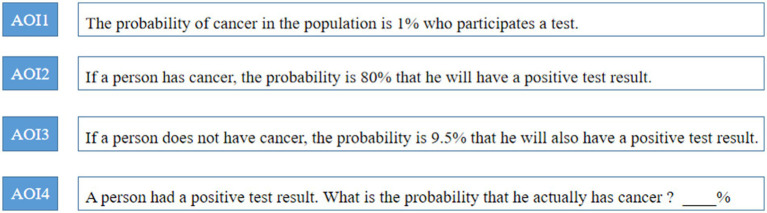
Areas of Interest of the Bayesian reasoning problem.

The entire material was segmented into five distinct areas of interest (AOIs) (see Figure 1). The sentence pertaining to the base rate was designated as AOI1 (area: 61642), while the hit rate sentence was identified as AOI2 (area: 61642), and the false alarm rate sentence was labeled AOI3 (area: 68112). The sentence describing the problem itself was categorized as AOI4 (area: 124936), with the remaining regions falling under AOI5.

### Experimental instrument

The eye movement instrument utilized in this study was the Eye-Link II model, manufactured by SR Research company. This instrument has a data acquisition rate of 250 Hz, ensuring high precision in tracking eye movements. The average fixation position error is less than 0.5°, indicating accurate measurement of eye fixation positions. The spatial resolution of the instrument is less than 0.005°, enabling detailed tracking of eye movements. The reasoning materials were converted into an 800 × 600 pixel image, with a black background and white text. The text was presented in 21-point SimSun font (1.5 line spacing) on a black background. The task was displayed as an 800 × 600 pixel image centered on a 17-inch screen.

### Procedure

The subjects sat in a chair 70 cm away from the monitor, the instructions for the test were explained, and subjects were briefly introduced the experimental process. Participants were given the response apparatus and were told how to operate it. Then, they put on the eye cap, and their head was fixed in a relatively stable position; and we conducted calibration, validation calibration (nine o’clock) and drift calibration.

**Figure 2:**
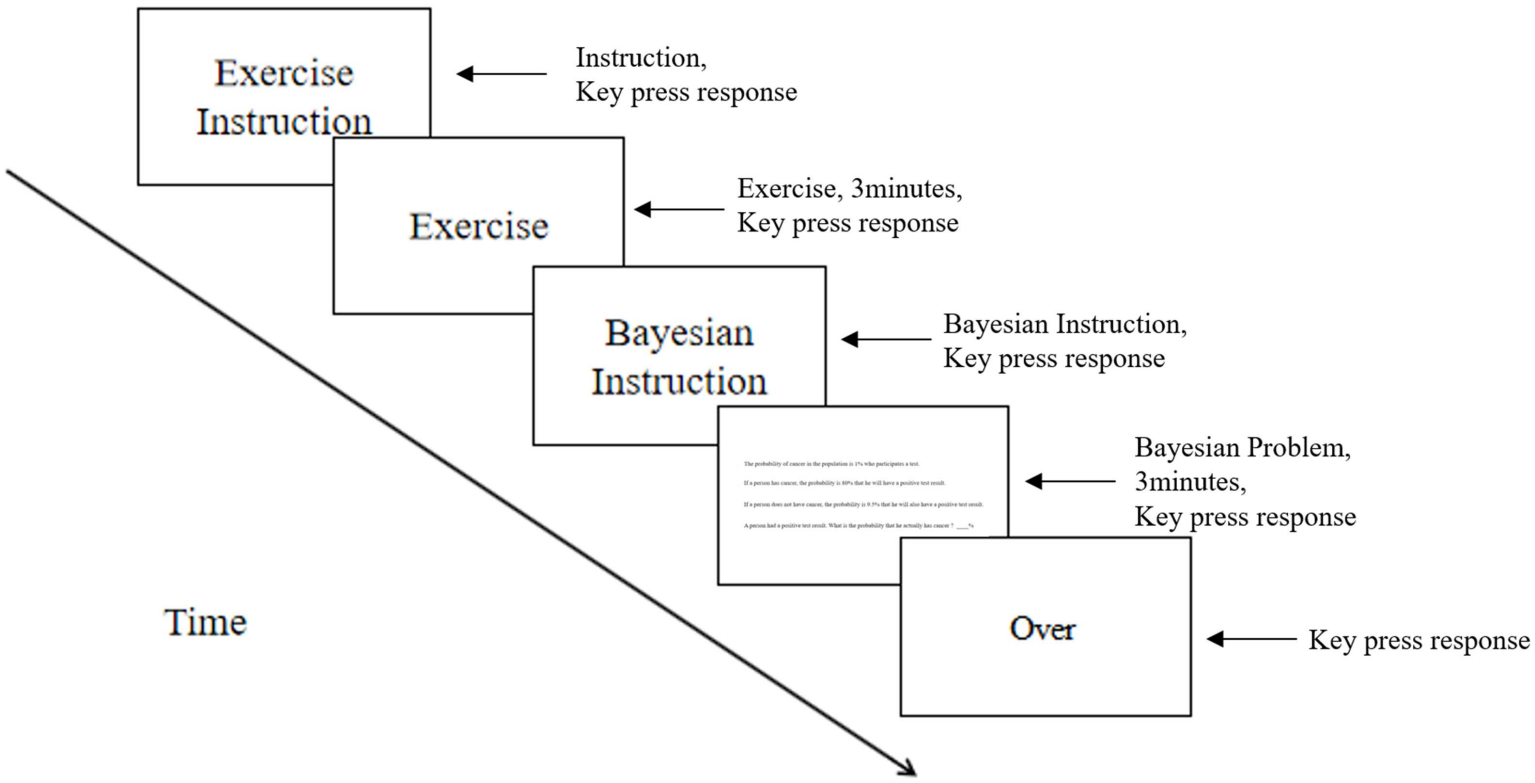
Flow chart of the eye movement procedure.

After drift calibration, the subjects were presented with instructions (all the instructions and problem materials were converted into pictures for presentation, and the text was presented in the center of the picture), practice exercises, confirmation instructions, Bayesian questions, and concluding remarks; the entire process is shown in Figure 2. The Bayesian reasoning task was presented in a randomized across. The duration of the eye movement task was approximately 10 min, and after the task, the subjects needed to complete the Embedded Figure Test (EFT) and fill in the basic data; the duration of the task was approximately 20 min. At the end of the experiment, the subjects were given a small gift.

## Results

### Global measures

#### Total fixation duration

Analysis of variance showed that the main effect of hit rate was significant in the index of total fixation duration (*p* < 0.05). When the hit rate was 50%, the total fixation duration was the lowest (39.85 ± 17.37 s), while when the hit-rate was 80%, it was the highest (58.54 ± 33.66 s) (Table 1).

**Table 1:** Overall eye movement patterns across different hit rates and cognitive style groups.

Hit-rate	Cognitive style	Total fixation duration (s)		Number of fixations	
		*M*	SD	*M*	SD
High	F-I	78.13	26.37	328.17	85.43
	F-D	47.86	33.31	191.36	113.44
Mid	F-I	34.10	11.12	157.00	49.45
	F-D	42.16	19.34	171.00	60.40
Low	F-I	39.03	19.06	177.00	81.79
	F-D	44.97	26.02	190.90	94.46

#### Number of fixations

Analysis of variance showed that the main effect of hit rate was significant for the index of the number of fixations (*p* < 0.05), and when the hit rate was 50%, the number of fixations was the lowest (167.00 ± 55. 97). The number of fixations was highest when the hit rate was 80% (239.65 ± 121.92). The interaction between hit rate and cognitive style was significant. Through simple effect analysis, the number of fixations of field-dependent participants at different hit rates were different (*F*(2, 13) = 8.072, *p* = 0.005), and their fixation frequency at a high hit rate level was significantly higher than that at a medium level (*p* = 0.004) or low level (*p* = 0.005).

### Local measures

#### Area of interest

From the analysis of the global measures, it was found that there are differences in the fixation duration and the number of fixations of the inference at different hit rate levels. To further explore the base rate and hit rate of the inference at each hit rate level, whether there is a difference in the degree of attention between the false alarm rate and the problem information, further analysis in the interest area is needed (Table 2).

**Table 2:** The total fixation time (s) of the reasoner in the areas of interest W1-W4.

Hit-rate	Cognitive style	W1	W2	W3	W4
High	F-I	10.71	22.037	20.225	16.410
	F-D	9.263	14.529	11.873	8.904
Mid	F-I	7.391	10.623	7.075	5.944
	F-D	6.678	12.026	9.852	9.466
Low	F-I	5.988	10.409	11.465	8.384
	F-D	7.903	13.640	12.428	9.402

To carefully study whether different categories of reasoners pay attention to various types of information differently under different conditions, all of the material was divided into 5 areas of interest (AOI), with AOI 1 contains the base rate, AOI 2 including the hit rate, AOI 3 containing the false alarm rate, AOI 4 containing problem information, and interest area 5 containing other areas. Since AOI 4 is an important area of interest requiring attention, the main analysis of the study focuses on AOI 4. The scope of each AOI is as follows.

The main results are as follows. (1) For the index of total fixation time, the difference between areas of interest was significant (*F*(3, 255) = 6.31, *p* < 0.01). LSD showed that AOI2 > AOI3 > AOI4 > AOI1. (2) For the index of the number of fixations, the difference between AOI was significant (*F*(3, 255) = 4.78, *p* < 0.01). LSD showed that AOI 2 > AOI 3 > AOI 4 > AOI 1.

Analysis of variance showed that the main effect of areas of interest was significant (*p* = 0.024 < 0.05) and the main effect of hit rate was significant (*p* = 0.001). The main effect of cognitive style was not significant, and the interaction between hit rate and cognitive style was significant (*p* = 0.002).

Further analysis of the interaction between hit rate and cognitive style revealed the following results: For field-independent individuals, there were differences in total fixation duration across the various AOIs (*F*(2.25, 63) = 4.588, *p* = 0.11 < 0.05), but no significant differences in total fixation duration at different hit rate levels (*F*(2, 28) = 0.181, *p* = 0.836). For field-dependent individuals, there were no significant differences in total fixation duration across the AOIs (*F*(1.75, 23) = 2.011, *p* = 0.161), but there were differences in total fixation duration at each hit rate level (*F*(2, 13) = 7.068, *p* = 0.008 < 0.01).

#### Proportion of fixation

To clarify the level of attention given to the area of interest throughout the entire process, this study divides the duration of the process into 20 equal intervals. The analysis of the time process allows us to more thoroughly examine the several stages of the inference process.

**Figure 3:**
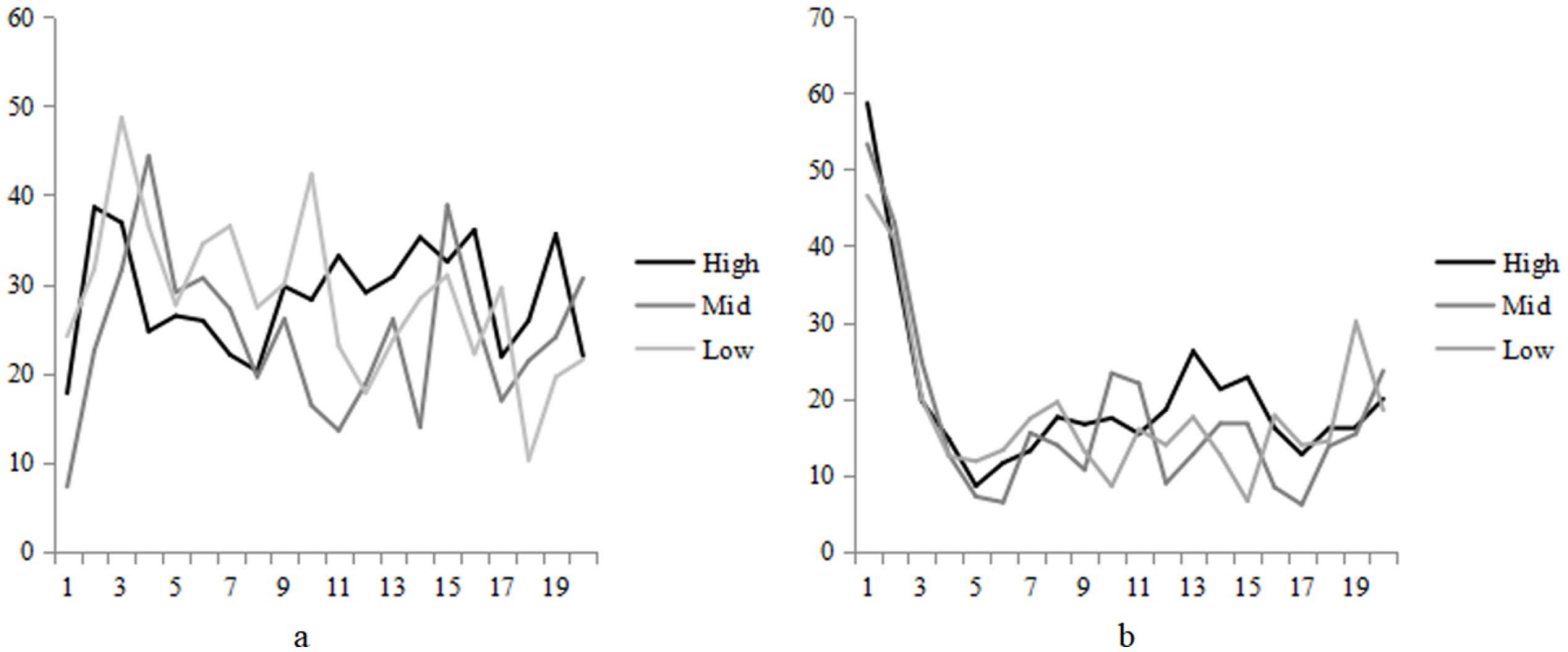
Proportion of fixation of the hit rate and cognition style [**(a)** for field-independent and **(b)** for field-dependent] in each stage of the reasoning process.

Based on the data from all subjects, the percentage of time spent fixated in each interest area was calculated, and the areas of interest, including the base rate, hit rate, false alarm rate and problem description, were compared in the same diagram, and the results are shown in the Figure 3.

Modulating the level of hit rate has an impact on attention toward the base rate during inference, primarily manifesting differences in the second and third stages, while no distinctions are observed in the first stage. Addressing the scenario of a high hit rate, attention toward the base rate reaches its peak in the initial phase of the second stage. When participants engage in Bayesian problem-solving, attention toward the base rate exhibits its peak in the middle of the second stage. Conversely, when tackling a low hit rate Bayesian problem, the peak of attention toward the base rate occurs in the third stage.

## General discussion

### Reasoning processing stage

Eye-movement allow for real-time recording of a viewer’s visual fixations, enabling exploration of the various processes involved by analyzing the overall duration of fixations during the inference process. In the problem-solving process, inference unfolds across three distinct stages (Shi et al., 2010).

The first stage entails the representation of the Bayesian reasoning problem. Reasoners’ attention toward each area of interest undergoes a turning point across the five designated areas. During the initial 25% of the inference process, problem information is preliminarily characterized. As the reasoning process unfolds, attention toward the base rate diminishes while greater focus is placed on the hit rate. Participants allocate comparable attention to the false alarm rate and the problem description information (Shi et al., 2010).

The second stage involves the integration of probability information and the selection of strategies. The middle 65% of the time is dedicated to establishing the relationship between probability information and selecting a probability estimation strategy. In the early part of this stage, emphasis on the problem description information and hit rate may lead to a deeper problem analysis, albeit at the expense of neglecting the base rate. In the middle of this stage, attention is relatively evenly distributed across different types of information, suggesting the subject’s intent to plan and integrate the information effectively. Toward the later part of this stage, the base rate tends to be relatively overlooked, with increased value placed on the hit rate (Shi et al., 2019).

The third and final stage, comprising the last 10% of the reasoning process, corresponds to the probability judgment stage. Overall analysis reveals that reasoners allocate equal attention to probability information during the decision-making stage and do not disregard the base rate (Shi et al., 2010; Shi et al., 2019).

### Ranking the importance of probability in Bayesian reasoning

The utilization of eye movement technology presents a significant advantage in that it allows for the recording of immediate information collection during inference, thereby enabling the reconstruction of the entire reasoning process based on real-time data acquired by the eye movement instrument.

Through the analysis of various eye movement indexes, it was found that the subject pays the most attention to the hit rate, followed by the false alarm rate and the problem description information, and it pays the least amount of attention to the base rate. The reasoner’s fixation time on the hit rate exceeds that on the base rate by approximately 50%, and the hit rate is indeed the most important information in the process of solving the problem. The subject also pays more attention to the false alarm rate, only less than the hit rate, perhaps because the problem description information contains two characteristics: illness|positive (p|h). These two characteristics are specifically included in the hit rate (p|h), and the false alarm rate is closely connected to these two characteristics of the system (−p|h). It is possible that the degree of attention paid to the information is affected by the degree of fit between them and the task information, and the higher the degree of fit is between them, the higher the level of attention the reasoner will allocate to them (Zhang et al., 2003).

Although the base rate exhibits the shortest fixation duration and frequency, it is noteworthy that participants do not entirely disregard it. However, the analysis of fixation duration and frequency merely indicates the extent to which the reasoner attends to each type of information, visually processes the information, and engages with it. This analysis does not address the extent to which the subject utilizes this information in making inference decisions or calculating the results. The relationship between the utilization of information and computation in decision-making is not directly examined. Therefore, further research utilizing Event-Related Potentials (ERPs) should be conducted to investigate the specific information processing mechanisms in the future (Shi et al., 2019).

### The mechanism of hit rate

Complex Bayesian reasoning and the hit rate play crucial roles in problem-solving, and variations in the hit rate level have a certain impact on the process.

Regarding the indices of total fixation duration and fixation count, there is a significant main effect of the hit rate. When solving Bayesian reasoning problems, the reasoning process exhibits the longest fixation duration when the hit rate is 80%, and the fixation duration is longer for reasoning problems with a hit rate of 1%. Conversely, when the hit rate is 50%, the fixation duration is the shortest. The fixation count follows a similar pattern, with high, low, and medium orders. However, there is no difference in the average fixation duration. Inference demonstrates the fewest fixations and the lowest number of guesses when the hit rate is 50%. This might be attributed to the ease of understanding inference at the 50% hit rate, as it aligns with common everyday experiences. Consequently, reasoners can express it quickly, recognizing that the hit rate itself constitutes the most critical information required for Bayesian reasoning. By simplifying the representation of the hit rate, the overall reasoning time is reduced (Tang and Shi, 2011).

The variation in hit rate levels influences the attention allocated to the base rate, primarily during the second and third stages, while no difference is observed in the first stage. When tackling problems with a high hit rate, there is an early-stage peak of attention directed toward the base rate. In contrast, when individuals solve Bayesian problems with a high hit rate, the peak attention toward the base rate emerges in the middle of the second stage. However, in scenarios involving low hit rates, the attention peak toward the base rate occurs in the third stage (Tang and Shi, 2011).

### Differences in information processing among people with different cognitive styles

Cognitive style refers to individuals’ preferred and habitual ways of organizing and interpreting information, encompassing their perceptual, memory, thinking, and problem-solving preferences. Field-dependent individuals rely on external environmental cues during information processing, whereas field-independent individuals rely on internal perceptual cues for information processing.

Analyzing the overall fixation duration during inference revealed that field-dependent subjects required more time to solve the problem compared to field-independent subjects. The index of the number of fixations also indicated a higher number of fixations among field-independent individuals compared to field-dependent individuals. Further analysis showed that the fixation frequency of field-dependent subjects was influenced by the hit rate, while field-independent subjects were not affected by it (Wang and Ouyang, 2004).

When examining eye movement indexes within each area of interest, it was found that fixation duration in each area of interest varied for field-independent individuals and was unaffected by the hit rate. However, for field-dependent individuals, there was no difference in fixation duration across each area of interest, but it was influenced by the hit rate. Overall, field-dependent participants exhibited shorter fixation times, and their fixation duration varied across different areas of interest, indicating unequal attention allocation during problem-solving and a strategy distinct from that of field-independent individuals. Field-independent individuals effectively allocate their resources and are not easily influenced by external cues such as changes in the hit rate.

In contrast, field-independent participants demonstrated longer overall fixation times with no difference in the distribution of time across each area of interest. This suggests that field-independent individuals take more time to solve the problem than field-dependent individuals, and they allocate equal attention to information within each area of interest without a distinction of priority. Additionally, the performance of field-dependent individuals is also influenced by changes in the hit rate, indicating that external environmental changes truly affect the reasoning time allocated by field-dependent individuals (Shi et al., 2010; Wang and Ouyang, 2004).

## Conclusion

This study elucidates critical cognitive mechanisms in Bayesian reasoning, emphasizing the interplay between attention allocation, cognitive style, and reasoning stages. Key findings include: (1) Attention prioritizes hit rate over false alarm and base rates, with insufficient focus on the latter leading to underestimation of its role; (2) Reasoning unfolds through problem representation, information integration/strategy selection, and probability judgment; (3) Hit rate dominates attention and influences reasoning time, effort, and base-rate focus; (4) Field-independent individuals exhibit superior attention allocation and analytical processing compared to field-dependent counterparts.

While this work advances understanding of Bayesian reasoning, several limitations warrant attention. First, reliance on a single task (breast cancer problem) may restrict generalizability to other contexts. Future studies should incorporate diverse Bayesian scenarios (e.g., financial or ecological decision-making) to validate robustness. Second, the college-student sample limits population representativeness; replications across age groups and educational backgrounds are needed. Third, individual differences in numeracy or statistical literacy—unmeasured here—may confound results. Incorporating these as covariates or control variables would strengthen causal claims.

Future research should extend findings to real-world applications, such as optimizing medical diagnostics or financial risk assessments, where Bayesian reasoning is critical. Additionally, exploring interactions between emotional/motivational factors (e.g., stress, incentives) and cognitive styles could deepen understanding of decision-making under uncertainty. Finally, integrating other cognitive dimensions (e.g., working memory, metacognition) may clarify their role in attention-resource allocation.

The findings underscore the value of tailoring educational interventions to cognitive styles. For instance, training field-dependent individuals to resist external cues or emphasizing base-rate salience could improve Bayesian reasoning. Eye-tracking technology, validated here as a powerful tool for mapping attention dynamics, offers promise for designing adaptive learning systems. By addressing these gaps, future work may bridge laboratory insights with real-world decision-making challenges, fostering more statistically literate societies.

## Data Availability

The raw data supporting the conclusions of this article will be made available by the authors, without undue reservation.
